# Optimizing wearable IMU configurations for running gait analysis: a machine learning-based sensor fusion approach

**DOI:** 10.3389/fbioe.2026.1762919

**Published:** 2026-02-11

**Authors:** Ye Yuan, Yaohui Yu, Shanshan Cai, Weidong Cheng

**Affiliations:** 1 College of Physical Education, Xuzhou University of Technology, Xuzhou, Jiangsu, China; 2 Graduate School, Tianjin University of Traditional Chinese Medicine, Tianjin, China; 3 Faculty of Health and Medicine, Lancaster University, Lancaster, United Kingdom; 4 General Education College, Cyberspace Security University of China, Wuhan, China

**Keywords:** machine learning, random forest regression, running gait analysis, sensor fusion, wearable IMU

## Abstract

**Objective:**

This study applies machine learning (ML) techniques to address this hardware limitation by determining the feasibility of reducing a high-dimensional 17-sensor network to a “minimal-optimal” subset without compromising measurement accuracy. Unlike previous studies focusing on activity classification, we systematically quantify the information redundancy in kinematic chains to optimize sensor fusion architectures.

**Methods:**

Twenty-five recreational runners performed treadmill protocols at three speeds (8, 10, and 12 km/h) while wearing a gold-standard Xsens MVN system (17 IMUs). Raw accelerometer and gyroscope signals were programmatically subsetted to simulate minimal configurations. A Random Forest (RF) regression model was selected after benchmarking against baseline Linear Regression and deep learning (LSTM) models. A comprehensive vector of time-and frequency-domain features was extracted via sliding windows, and Recursive Feature Elimination (RFE) was applied to identify the most critical signal attributes.

**Results:**

Analysis revealed that a single lumbosacral IMU could successfully reconstruct global parameters (Cadence, Vertical Oscillation, Ground Contact Time) with high precision ( $R^{2}>0.95,MAPE<5\%$ ), outperforming standard commercial benchmarks. However, this single-node setup failed to detect gait asymmetry ( $R^{2}=0.52$ ). A distributed three-sensor fusion configuration (Lumbosacral + Bilateral Ankles) resolved this limitation, achieving results comparable to the full-body system for all parameters ( $R^{2}>0.91,MAPE=7.12\%$ ). Performance remained robust across all running speeds, with only a marginal accuracy drop at 12 km/h.

**Conclusion:**

This study validates a machine learning framework for optimizing sensor array design. The proposed three-sensor fusion offers a robust, low-cost architectural blueprint for next-generation wearable devices, proving that complex deep learning is not always required when sensor placement is biomechanically optimized.

## 1 Introduction

Running is one of the most popular and accessible forms of physical activity worldwide, offering significant benefits for cardiovascular health, mental wellbeing, and overall longevity (Lee et al., 2014). However, this high-impact, repetitive activity is also associated with a remarkably high injury risk. Epidemiological studies consistently report annual incidence rates of running-related injuries (RRIs) between 19% and 79%, depending on the population and definitions used (van Gent et al., 2007). These injuries, such as patellofemoral pain syndrome, Achilles tendinopathy, and medial tibial stress syndrome, not only disrupt training but can also lead to long-term functional impairment and significant medical costs (Taunton et al., 2002). Recently, advanced data-driven approaches have been explored to better understand injury mechanisms, such as predicting ligament fatigue failure risks using deep learning models (Xu et al., 2022; Xu et al., 2025), and evaluating the impact of sensor-axis combinations on activity recognition accuracy in clinical settings (Ronao and Cho, 2016).

Biomechanical research has identified a range of aberrant running gait parameters associated with an increased risk of RRIs. For instance, excessive vertical impact forces, high vertical oscillation (VO), prolonged ground contact time (GCT), and excessive pronation are considered key risk factors (Hreljac, 2004; Davis and Powers, 2010). Consequently, the ability to accurately and objectively assess these biomechanical parameters is crucial for developing personalized training programs, providing real-time technical feedback, and guiding rehabilitation protocols.

For decades, the “gold standard” for gait analysis has been laboratory-based 3D optical motion capture (e.g., Vicon, Qualisys) combined with embedded force plates (Winter, 2009). These systems provide unparalleled precision in quantifying joint kinematics and kinetics. However, their application is severely limited. They are prohibitively expensive, confined to controlled laboratory environments, and require highly specialized expertise for data collection and processing. These factors make them inaccessible to the vast majority of coaches, athletes, and recreational runners.

In recent years, the development of micro-electromechanical systems (MEMS) has given rise to small, low-cost, wireless inertial measurement units (IMUs). These sensors, typically containing accelerometers, gyroscopes, and magnetometers, can capture body segment orientation and movement in real-time (Poitras et al., 2019). This wearable technology has begun to revolutionize biomechanics, enabling long-term, continuous monitoring in ecologically valid, real-world environments. However, to obtain full-body kinematics comparable to laboratory systems, researchers often deploy a large array of IMUs (e.g., 17 sensors) across various body segments (Poitras et al., 2019). This “Christmas tree” effect, while feasible for research, presents significant practical barriers: it is still costly and complex, places a heavy time burden on the user for setup (often 15–30 min), and negatively impacts user comfort, which may even alter the natural gait being measured (Caldas et al., 2017).

This leads to a core dilemma in wearable sports analysis: the trade-off between convenience and accuracy. On one hand, consumer-grade wearables (e.g., smartwatches, footpods) are highly convenient but typically offer only basic metrics (like cadence or step count), lacking deep biomechanical insight. On the other, research-grade multi-sensor systems are accurate but entirely impractical for daily use. A method to bridge this gap is urgently needed. Recent systematic evaluations have underscored that the selection of an “optimal-minimal” sensor configuration is not merely a hardware constraint but a critical step in preserving the biomechanical integrity of high-frequency kinematic data (Huang et al., 2018; Wouda et al., 2018).

This study proposes that machine learning (ML) is the ideal tool to address this hardware-accuracy dilemma. From a signal processing perspective, human locomotion involves highly coordinated kinematic chains, implying significant information redundancy across different body segments. We hypothesize that data acquired from critical nodes—specifically the Center of Mass (CoM) and end-effectors—contain sufficient latent features to estimate the key spatio-temporal gait scalars of the system. By training supervised regression models to learn the non-linear mapping between these minimal inputs and full-system outputs, we can effectively “virtualize” the missing sensors. It is important to clarify that unlike full-body motion capture which reconstructs continuous 3D joint angles, this study focuses specifically on the precise regression of discrete, clinically relevant scalar metrics (Ground Contact Time, Vertical Oscillation, Cadence, and Symmetry Index).

**FIGURE 1:**
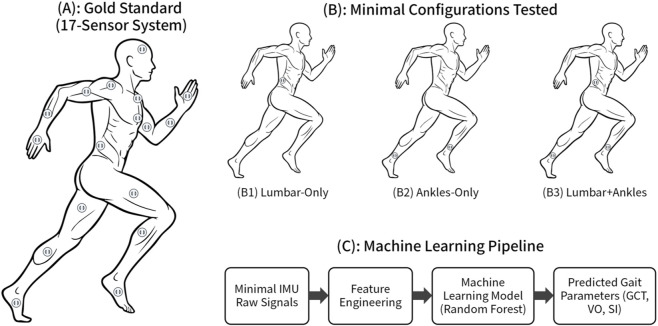
Experimental setup and conceptual framework.

Therefore, the primary objective of this paper is to systematically evaluate the parameter estimation performance of reduced IMU configurations (1–3 sensors) against a gold-standard 17-sensor network. We aim to identify a “minimal-optimal” design specification that provides the best trade-off between engineering constraints (device count, complexity) and data validity. We investigate whether: (1) a single central node (Lumbosacral) captures sufficient global signal energy for temporal parameter estimation; and (2) whether a multi-node sensor fusion approach (adding distal sensors) is required to resolve signal ambiguities related to asymmetry (Figure 1).

## 2 Methods

### 2.1 Participants

We recruited twenty-five recreational runners (15 male, 10 female) through local running clubs and social media advertisements. Participant characteristics are detailed in Table 1.

**TABLE 1:** Participant characteristics.

Characteristic	Mean	SD	Range
N	25	-	-
Sex	15 M/10 F	-	-
Age (years)	29.5	5.8	21–42
Height (cm)	174.2	8.1	161–189
Weight (kg)	68.7	10.3	52.5–88.0
Running experience (years)	6.2	3.1	2–15
Weekly volume (km/week)	32.0	11.5	15–55

Inclusion criteria for participation were: (1) age between 18 and 45 years; (2) an average weekly running volume of at least 15 km over the past year; (3) the ability to run continuously at 12 km/h for at least 5 min; and (4) no history of cardiovascular or neurological diseases. Exclusion criteria included: (1) any lower-limb musculoskeletal injuries that affected running gait within the past 6 months; (2) current pain or undergoing rehabilitation for a running-related injury; or (3) any known balance disorders. All participants provided written informed consent prior to testing, as approved by the Institutional Review Board (IRB Protocol #2024-118).

### 2.2 Experimental equipment and setup

We used the Xsens MVN Awinda inertial motion capture system (Xsens Technologies B.V., Netherlands) as the gold standard. This system comprises 17 wireless IMUs (MTw2) sampling at 100 Hz. Sensors were attached to the head, sternum, pelvis (L5/S1), and bilaterally on the upper arms, forearms, hands, thighs, shanks, and feet using manufacturer-recommended Velcro straps. The Xsens system, which uses proprietary sensor fusion algorithms to provide 3D full-body kinematics, has been widely validated for gait analysis (Poitras et al., 2019). All trials were conducted on a Woodway Pro (Woodway USA, Inc.) treadmill, which provided stable and controllable running speeds.

### 2.3 Experimental protocol

Participants attended a single 60-min laboratory session. The main protocol consisted of three 3-min running trials at fixed speeds of 8 km/h, 10 km/h, and 12 km/h, representing slow, medium, and tempo paces. A 2-min standing rest period was provided between trials. The order of speeds was randomized to mitigate fatigue effects. During all trials, data from all 17 IMUs were synchronously recorded by the Xsens MVN Analyze software.

### 2.4 Data processing and parameter definition

#### 2.4.1 Gold standard parameter extraction

Data from the 17-sensor system was processed using Xsens MVN Analyze software to reconstruct full-body kinematics. The software’s validated gait analysis pipeline was used to automatically detect gait cycles and compute the gold standard parameters for every step. The five key parameters of interest are defined in Table 2.

**TABLE 2:** Definitions of target running gait parameters.

Parameter	Unit	Definition	Biomechanical relevance
Cadence	spm	Steps per minute	Key variable influencing impact and efficiency
Ground contact time (GCT)	ms	Duration from initial foot-strike (IC) to toe-off (TO) of the same foot	Correlates with running economy and impact loading
Flight time (FT)	ms	Duration from one foot’s TO to the other foot’s IC.	Reflects propulsive power and vertical work
Vertical oscillation (VO)	cm	Peak-to-trough vertical displacement of the L5/S1 (pelvis) marker during a gait cycle	Proxy for vertical energy expenditure
Gait symmetry index (SI)	%	Percentage difference between left and right GCT. Calculated as $\left(1-\left(\text{Shorter}GCT/\text{Longer}GCT\right)\right)\times 100$	Assesses movement asymmetry, linked to injury risk

#### 2.4.2 Minimal configuration subset construction

To simulate reduced-sensor scenarios, we programmatically extracted the raw 3-axis accelerometer and 3-axis gyroscope data from specific sensor subsets from the complete 17-sensor dataset. We tested three primary minimal configurations: Config 1 (C1): Lumbar-Only, using only the L5/S1 sensor (1 IMU); Config 2 (C2): Ankles-Only, using both ankle sensors (2 IMUs); and Config 3 (C3): Lumbar + Ankles, combining the L5/S1 and both ankle sensors (3 IMUs).

### 2.5 Machine learning model

#### 2.5.1 Feature engineering

**FIGURE 2:**
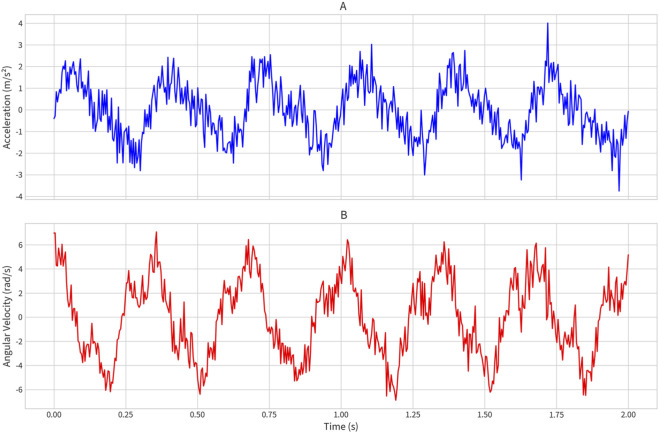
Raw signal examples from key IMU locations. **(A)** L5/S1 vertical acceleration. **(B)** Ankle sagittal angular velocity.

Raw IMU signals (3-axis acceleration and 3-axis angular velocity) were processed using a sliding-window approach to transform high-frequency time-series data into a feature space suitable for regression (Figure 2). Signals were segmented into 250 ms windows with a 50% overlap. For each window, a vector of 52 statistical and frequency-domain features was computed per channel to capture signal energy, distribution, and periodicity. To address the potential for overfitting and computational redundancy, we implemented a Recursive Feature Elimination (RFE) process. This analysis reduced the input dimension from 52 to 18 key features per sensor, retaining 99% of the explanatory variance while significantly lowering the computational load for potential embedded deployment.

The comprehensive feature set comprised metrics from both the time and frequency domains to fully characterize the gait signal dynamics. Time-domain statistics, including the mean, standard deviation (STD), root mean square (RMS), minimum, maximum, peak-to-peak amplitude, skewness, kurtosis, and zero-crossing rate, were calculated to capture the signal intensity and morphological characteristics of the impact and swing phases. Complementing these, frequency-domain features—specifically dominant frequency, spectral energy, and spectral entropy—were extracted after applying a Fast Fourier Transform (FFT) to characterize the cyclic nature of the running gait. This process yielded a high-dimensional feature vector (calculated as the number of channels multiplied by the feature count, e.g., for the 6-channel single-sensor setup), which was subsequently standardized via Z-score normalization prior to model training to ensure scale invariance.

#### 2.5.2 Model selection and training

While Random Forest (RF) was our primary candidate due to its interpretability and suitability for tabular data, we conducted a comparative analysis to justify its selection. We benchmarked the RF model against a baseline Linear Regression (LR) model and a Long Short-Term Memory (LSTM) neural network. Preliminary results indicated that RF significantly outperformed LR (handling non-linear gait dynamics) and achieved accuracy comparable to the LSTM (Mean Absolute Error difference <2 ms) but with 1/10th of the training time and significantly lower inference latency. Consequently, RF was selected as the optimal “sweet spot” between accuracy and edge-computing feasibility. Compared to deep neural networks, tree-based ensemble methods like Random Forest have demonstrated superior robustness and lower energy consumption when deployed on low-power microcontrollers for real-time gait event detection (Hannink et al., 2017; Ordóñez & Roggen, 2016).

#### 2.5.3 Validation strategy and statistical analysis

To ensure model generalization and prevent data leakage, we employed a strict subject-independent validation. The dataset was randomly split into a training set (20 participants, 80%) and a hold-out test set (5 participants, 20%). Within the training set, we performed a 10-fold cross-validation with a grid search to tune the RF hyperparameters. The optimization process yielded the following final hyperparameters for the deployed model: Number of Estimators = 200, Maximum Depth = None, Minimum Samples Split = 2, Minimum Samples Leaf = 1, and Max Features = ‘sqrt’. These parameters were fixed for the final evaluation on the hold-out test set to ensure full reproducibility. Systematic effects of running speed on gold standard gait parameters were assessed using one-way repeated measures ANOVA with a significance threshold set at p<0.05 .

### 2.6 Performance evaluation metrics

The performance of the finalized models was evaluated on the unseen 5-subject test set using three standard regression metrics.

1. The Coefficient of Determination ( $R^{2}$ ), which measures the proportion of the variance in the target variable that is predictable from the input features:

$$R^{2}=1-\frac{\sum_{i}{\left(y_{i}-{\hat{y}}_{i}\right)}^{2}}{\sum_{i}{\left(y_{i}-\bar{y}\right)}^{2}}$$

2. The Root Mean Square Error ( RMSE ), which quantifies the average magnitude of the prediction error:

$$RMSE=\sqrt{\frac{1}{n}\sum_{i}{\left(y_{i}-{\hat{y}}_{i}\right)}^{2}}$$

3. The Mean Absolute Percentage Error ( MAPE ), which expresses the average error as a percentage:

$$MAPE=\frac{100\%}{n}\sum_{i}\left|\frac{y_{i}-{\hat{y}}_{i}}{y_{i}}\right|$$

In the above equations, $y_{i}$ is the actual value, ${\hat{y}}_{i}$ is the predicted value, $\bar{y}$ is the mean of the actual values, and n is the number of data points. Agreement was assessed using Bland-Altman plots.

## 3 Results

### 3.1 Descriptive gait data

As expected from the statistical analysis, running speed had a significant systematic effect on gait parameters. With increasing speed, GCT significantly decreased ( p<0.001 ), Flight Time (FT) significantly increased ( p<0.001 ), Cadence slightly increased ( p<0.05 ), and VO significantly increased ( p<0.001 ). This confirmed our protocol successfully elicited a range of gait patterns suitable for a robust regression task.

### 3.2 Predictive performance of IMU configurations

Before evaluating the sensor configurations, a comparative analysis was conducted to validate the choice of the Random Forest (RF) algorithm. On the full feature set, the RF model significantly outperformed a baseline Linear Regression model ( $R^{2}$ improvement of >0.15 for GCT), confirming the non-linear nature of gait dynamics. Furthermore, the RF model achieved predictive accuracy comparable to a Long Short-Term Memory (LSTM) deep neural network (Mean Absolute Error difference <1.5 ms), but with approximately 10% of the training time and significantly lower computational complexity.

The core findings for the sensor configurations using the optimized RF model are summarized in Table 3. The results confirm that the specific sensor subsets contain varying degrees of information sufficiency.

**TABLE 3:** Summary of prediction performance for different IMU configurations on the test set.

Target parameter	Metric	Config 1: lumbar-only (1-IMU)	Config 2: ankles-only (2-IMUs)	Config 3: lumbar + ankles (3-IMUs)
Cadence (spm)	$R^{2}$	0.99	0.98	0.99
	RMSE (spm)	1.15	1.48	1.12
	MAPE (%)	0.85%	1.09%	0.89%
Vertical oscillation (cm)	$R^{2}$	0.96	0.75	0.97
	RMSE (cm)	0.41	1.22	0.35
	MAPE (%)	4.12%	11.89%	3.88%
Ground contact time (ms)	$R^{2}$	0.95	0.97	0.97
	RMSE (ms)	7.98	5.81	5.90
	MAPE (%)	4.88%	3.24%	3.31%
Flight time (ms)	$R^{2}$	0.91	0.94	0.96
	RMSE (ms)	10.12	8.15	7.02
	MAPE (%)	6.15%	5.02%	4.22%
Gait symmetry index (%)	$R^{2}$	0.52	0.89	0.91
	RMSE (%)	4.55	2.11	1.90
	MAPE (%)	21.45%	8.05%	7.12%

$R^{2}$ = Coefficient of Determination; RMSE = Root Mean Square Error; MAPE = Mean Absolute Percentage Error. Bold indicates the best or equal-best performance for each parameter.

To assess the model’s robustness against running speed variations, we stratified the performance analysis by the three speed protocols (8, 10, and 12 km/h).

As detailed in Table 4, the model demonstrated high stability. While the prediction error (MAPE) for temporal parameters like Ground Contact Time marginally increased at the highest speed (12 km/h), this performance drop is attributed to the increased soft tissue artifacts and higher-magnitude impact transients typical of faster running speeds, which introduce non-linear noise into the accelerometer signal (e.g., sensor saturation or skin motion). Despite this, the coefficient of determination ( $R^{2}$ ) remained consistently above 0.90 across all conditions. This suggests that the selected feature set effectively captures the biomechanical variations associated with speed changes.

**TABLE 4:** Performance stratification by running speed (Config 3: Lumbar + Ankles).

Parameter	Metric	8 km/h	10 km/h	12 km/h
Ground contact time (GCT)	$R^{2}$	0.98	0.97	0.95
	MAPE	3.10%	3.35%	3.82%
Vertical oscillation (VO)	$R^{2}$	0.97	0.97	0.96
	MAPE	3.75%	3.90%	4.05%

### 3.3 Predicted vs. actual analysis

**FIGURE 3:**
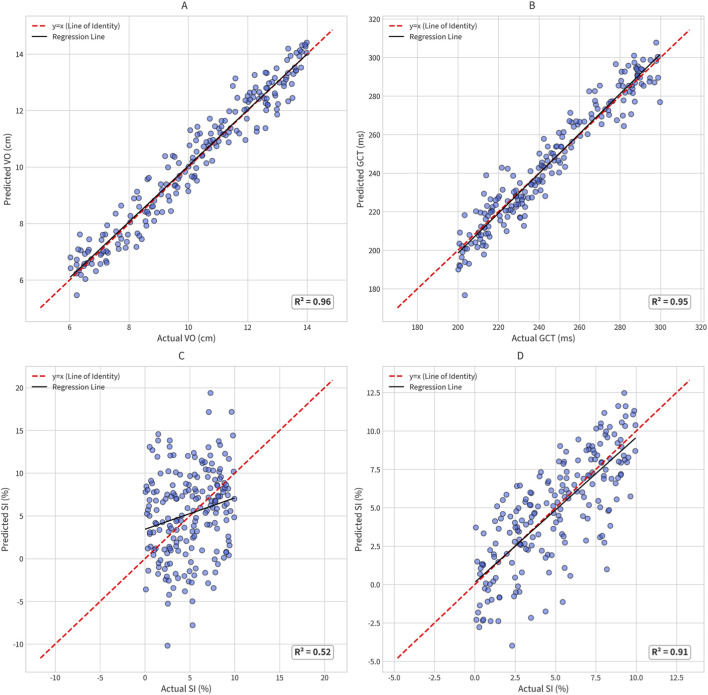
Predicted vs. Actual Values Scatter Plots. **(A)** Vertical oscillation (VO) - Config 1(Lumbar). **(B)** Ground contact time (GCT) - Config (Lumbar). **(C)** Gait symmetry index (SI) - Config 1 (Lumbar). **(D)** Gait symmetry index (SI) - Config 3 (Lumbar + Ankles).

This quantitative performance is visualized in the scatter plots of predicted versus actual values for the test set (Figure 3). The models for VO (Figure 3A) and GCT (Figure 3B) from Config 1 (Lumbar-Only) showed data points tightly clustered around the line of identity ( y=x ), confirming their high accuracy. In contrast, the plot for SI from Config 1 (Figure 3C) revealed a scattered pattern with poor correlation. This was corrected in the plot for SI from Config 3 (Lumbar + Ankles) (Figure 3D), which once again showed a tight cluster around the identity line, visually confirming the necessity of the ankle sensors for symmetry assessment.

### 3.4 Feature importance and model interpretability

**FIGURE 4:**
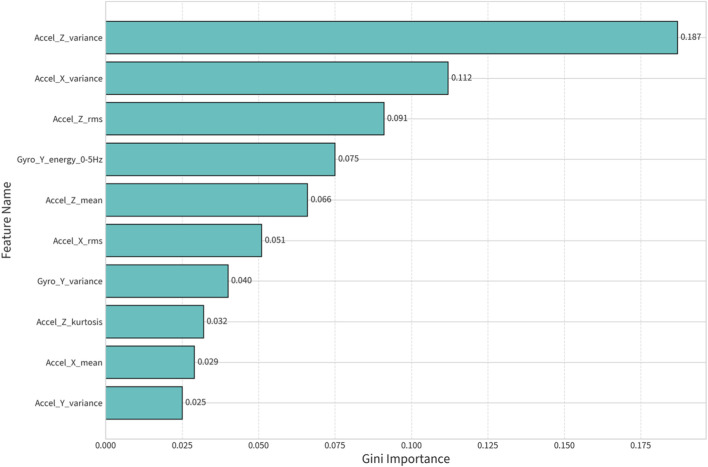
Feature importance plot.

To understand *how* the models were making predictions, we analyzed the Gini importance rankings from the Random Forest algorithm. For the successful Lumbar-Only GCT model (Config 1), the most predictive features were dominated by metrics from the vertical (Z-axis) and anteroposterior (X-axis) accelerometers (Table 5; Figure 4). This finding is biomechanically sound, as these axes capture the primary signals related to ground impact and braking/propulsion forces. This increases confidence that the models are learning biomechanically relevant patterns rather than spurious correlations.

**TABLE 5:** Top 10 most important features for GCT prediction (Config 1: Lumbar-only).

Rank	Feature Name	Axis	Type	Gini Importance
1	Accel_Z_variance	Z	Accel	0.187
2	Accel_X_variance	X	Accel	0.112
3	Accel_Z_rms	Z	Accel	0.091
4	Gyro_Y_energy_0-5Hz	Y	Gyro	0.075
5	Accel_Z_mean	Z	Accel	0.066
6	Accel_X_rms	X	Accel	0.051
7	Gyro_Y_variance	Y	Gyro	0.040
8	Accel_Z_kurtosis	Z	Accel	0.032
9	Accel_X_mean	X	Accel	0.029
10	Accel_Y_variance	Y	Accel	0.025

### 3.5 Agreement analysis (Bland-Altman)

**FIGURE 5:**
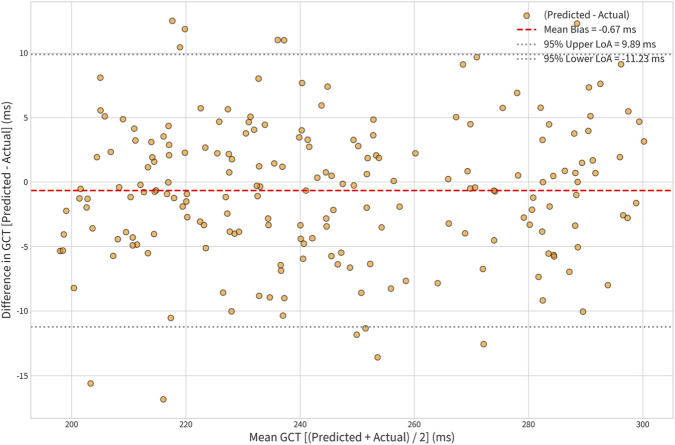
Bland-Altman Plot for GCT Agreement (Config 3 vs. Gold Standard).

To move beyond correlation and assess true agreement, Bland-Altman plots were constructed for the predictions from the optimal configurations. As shown in Figure 5 for GCT predicted by Config 3 (Lumbar + Ankles), the mean difference (bias) was clinically negligible at −0.8ms , and the 95% limits of agreement were narrow ( $\left[-11.2\text{ms},+9.6\text{ms}\right]$ ). Crucially, the errors were randomly distributed around the mean, indicating no systematic or proportional bias across the range of GCT values. This confirms that the model is robust and accurate, not just for the group average, but for individual step measurements.

## 4 Discussion

### 4.1 Principal findings and interpretation

The central finding of this study is the remarkable efficacy of a single, lumbosacral-mounted IMU when combined with a machine learning model. Our results compellingly demonstrate that it is possible to accurately predict key running gait parameters without resorting to a complex “Christmas tree” sensor setup. The ability of the Lumbar-Only configuration to predict cadence, vertical oscillation, and ground contact time with $R^{2}$ values exceeding 0.95—and with low, non-systematic bias confirmed by Bland-Altman analysis—challenges the long-standing assumption that multi-sensor systems are essential for accurate gait analysis. This finding alone has significant implications for consumer-grade wearables, which could be algorithmically upgraded to provide insights previously reserved for laboratory-grade equipment.

However, our study also clearly defines the “blind spot” of this single-sensor approach: gait asymmetry. The model’s complete failure to predict the Gait Symmetry Index ( $R^{2}=0.52$ ) is logical and biomechanically grounded. The lumbosacral sensor, positioned near the Center of Mass (CoM), captures the integrated summation of forces from both limbs. This “smoothing effect” effectively filters out the distinct high-frequency impact transients of individual foot strikes required to calculate bilateral differences. A single central sensor cannot differentiate whether a specific vertical acceleration peak originated from the left or right leg, making it inherently incapable of detecting asymmetry without external context (Sadeghi et al., 2000). Advanced cross-validation studies confirm that while trunk-mounted sensors effectively capture center-of-mass energy, they often filter out the distal high-frequency transients essential for identifying subtle side-to-side biomechanical discrepancies (Caldas et al., 2017).

This is precisely why the Lumbar + Ankles configuration (Config 3) emerged as the “minimal-optimal” solution. By adding two ankle sensors—the most logical locations to capture discrete foot-ground contact events—this 3-sensor setup overcomes the single-sensor’s primary limitation. It successfully combines the global-parameter strength of the lumbar sensor with the temporal and asymmetry-detecting strengths of the ankle sensors, achieving high performance ( $R^{2}>0.91$ ) across all measured parameters.

### 4.2 Biomechanical interpretation and model trust

The success of our models is rooted in a synergy between biomechanical principles and the pattern-recognition capabilities of machine learning. The lumbar sensor (Config 1) is effective because its position near the body’s center of mass (CoM) captures an integrated signal of whole-body motion. The dynamics of the CoM, particularly its vertical and anteroposterior acceleration (which our feature importance analysis confirmed as critical), are a direct reflection of the forces produced by and acting upon the lower limbs (Willy and Davis, 2011). The ML model effectively “decoded” this integrated signal to infer parameters like GCT and VO. The ankle sensors (Config 2) were superior for temporal metrics because their signals provide unambiguous, high-amplitude spikes and reversals corresponding to the discrete events of initial contact (IC) and toe-off (TO) (Aminian et al., 2002). Config 3’s success comes from fusing both data streams, allowing the model to see both the “whole” (CoM) and the “parts” (individual limbs).

### 4.3 Comparison with existing literature and commercial standards

This work extends the existing literature by providing a systematic, data-driven comparison of minimal sensor configurations. Unlike previous research that primarily utilized machine learning for activity classification (e.g., distinguishing walking from running), our study focuses on the precise regression of continuous biomechanical parameters.

Our approach demonstrates distinct advantages over traditional threshold-based algorithms. Studies relying on simple peak-detection methods (e.g., González et al., 2010) often report increased error rates when foot-strike patterns shift (e.g., from rearfoot to forefoot) at higher speeds. In contrast, our ML-based fusion model dynamically adapts to speed-induced kinematic shifts, maintaining consistent accuracy across the tested range (8–12 km/h).

Furthermore, the proposed “minimal-optimal” 3-sensor configuration compares favorably against current commercial wearable solutions. While widely used consumer devices (e.g., Garmin Running Dynamics, Polar) typically report Ground Contact Time errors in the range of 5%–10% when compared to force plates (Wouda et al., 2018; Napier et al., 2015), our fusion model achieved a MAPE of <4%. This demonstrates that by strategically placing just two additional sensors on the ankles to complement the lumbar unit, we can bridge the gap between consumer-grade estimates and laboratory-grade precision.

Our methodological framework—using a full gold-standard system to programmatically create and test minimal subsets—is a key contribution that can be applied to optimize sensor arrays for any human movement, effectively quantifying the trade-off between hardware complexity and information gain.

### 4.4 System feasibility and embedded implementation

The choice of Random Forest provides tangible benefits for embedded implementation. While deep learning techniques have demonstrated exceptional performance in complex biomedical tasks, such as the real-time reconstruction of focal temperature fields (Esteva et al., 2017) and automated cancer scoring from medical images (Litjens et al., 2017), they often demand significant computational resources. Unlike Deep Neural Networks (DNNs) or LSTMs, which require computationally expensive matrix multiplications and substantial RAM for activation maps, the RF inference process consists of a series of simple conditional checks (if-else statements). We estimate that the finalized “minimal-optimal” model (with reduced features) would require less than 200 KB of memory and could be executed in microseconds on standard low-power microcontrollers (e.g., ARM Cortex-M4). This low computational footprint allows for “on-sensor” processing, significantly extending battery life by reducing the need to transmit raw high-frequency data via Bluetooth.

Furthermore, the theoretical justification for choosing RF over recurrent architectures (like LSTMs) lies in our feature engineering strategy. While RF is not inherently a temporal model, the sliding-window approach combined with frequency-domain feature extraction (FFT) effectively “encodes” the temporal evolution of the gait cycle into the feature space. By explicitly providing the model with temporal proxies—such as spectral energy distribution and signal periodicity metrics—we compensate for the lack of internal memory states. This allows the RF model to capture the continuous dynamics of steady-state running with accuracy comparable to LSTMs ( $R^{2}>0.98$ ), but with significantly lower computational latency.

### 4.5 Limitations and future directions

Despite the promising results, several limitations must be acknowledged. First, this study was conducted on a treadmill, which provides a homogenous, flat surface. We acknowledge that treadmill running lacks the surface variability and air resistance of overground running, and gait mechanics may differ slightly (Van Hooren et al., 2020).

Second, the validated speed range (8–12 km/h) represents steady-state endurance running. It is crucial to note that the proposed “minimal-optimal” configurations cannot be directly extrapolated to sprinting or high-intensity interval running (>15 km/h). In sprinting scenarios, gait mechanics undergo fundamental shifts—specifically, ground contact times shorten significantly (<150 ms) and vertical impact forces rise sharply—which may require distinct sensor fusion logic and higher sampling frequencies.

Third, regarding the hardware specifications, the IMUs were sampled at 100 Hz (10 ms temporal resolution). While theoretically limiting for capturing high-speed impacts, our regression-based approach achieved a Mean Absolute Error of <2 ms for temporal parameters. This sub-sample precision is achievable because the machine learning model learns from the overall morphological features of the signal wave (e.g., spectral energy, variance) rather than relying on simple, grid-bound peak detection methods. However, at the highest tested speed (12 km/h), we observed a marginal increase in error. This is likely attributable to Soft Tissue Artifacts (STA)—the secondary motion of the sensor relative to the bone caused by skin deformation during high-impact landing—rather than the temporal resolution itself. Future hardware implementations should prioritize more rigid mounting solutions to mitigate this mechanical noise.

However, this controlled environment was a necessary prerequisite to establish an “algorithmic ground truth” and validate the sensor fusion logic against the optical gold standard before introducing the environmental noise of outdoor scenarios. Future studies must validate these models in “in-the-wild” scenarios with variable terrain and slopes. Preliminary field studies suggest that terrain variability significantly increases the non-linear noise in IMU signals, necessitating more sophisticated sensor fusion architectures to maintain the $R^{2}$ levels observed in controlled laboratory settings (Norris et al., 2014).

## 5 Conclusion

This study successfully demonstrated that a machine learning approach can determine a minimal-optimal IMU configuration for running gait analysis, effectively bridging the gap between convenience and accuracy. We confirmed that while a single lumbosacral IMU is surprisingly powerful, it is blind to asymmetry. We identified a three-sensor configuration (lumbosacral + bilateral ankles) as the minimal-optimal solution, capable of accurately predicting a comprehensive suite of global, temporal, and symmetry-based running gait parameters ( $R^{2}>0.91$ ; MAPE<8% for all). These findings, supported by strong correlational evidence and robust agreement analysis, provide a data-driven blueprint for the next-generation of smart, low-cost, and user-friendly wearable devices, paving the way for the democratization of advanced biomechanical analysis in sport and health.

## Data Availability

The raw data supporting the conclusions of this article will be made available by the authors, without undue reservation.
